# The Views and Aims o the Hospital Almoners' Council

**Published:** 1920-11-06

**Authors:** 


### HOSPITAL SOCIAL SERVICE.

### The Views and Aims o the Hospital Almoners' Council.

To readers of this paper the idea is familiar, but even to the uninitiated it needs but a moment's reflection to realise that the service rendered by the medical staff to the patients who flock to hospitals is not, and cannot be expected to be, adequate to their needs. The individual patient at the hospital appears for the moment as an isolated social unit with some bodily ailment which needs correction, but the very thing which brings him there is upsetting the balance of his social environment and making fresh difficulties in already difficult lines. The doctor cannot attempt to deal with these added causes of friction, nor can he hope to oversee the provision of the change of work, the sojourn in the country, the surgical instrument, or the special diet, to mention only one or two of the things which he may find it necessary to prescribe. Modern treatments, too, are often elaborate and require explanation, or a patient needs persuasion to adopt an urgently necessary plan, and time presses in the rush of an out-patient department. Moreover, a hospital is one charitable agency in a district where there are many others, where co-operation for the good of the individual must exist if waste of effort and failure to secure the best possible is to emerge. It is also one medical service among many, and all these need mutual consideration and linking up if harmonious working is to be attained.

A consideration of these things shows so clearly the need for someone within the hospital whose business they are, that it is to be wondered that the conception of a Social Service Department did not come sooner. Come eventually, however, it did. First in one hospital and then another, women were appointed to whom was given the perhaps slightly misleading title of almoner. America, with its characteristic readiness to adopt new ideas, has perhaps gone faster and further in the provision of its hospital social service, but even in this country progress has been steady. Too much attention was at first directed to one merely of the almoner's functions—the exclusion, namely, of the unsuitable recipient of charitable help, but this, which at first appealed strongly to the governing bodies, owing in part to aroused public opinion, has now fallen into its rightful place as a comparatively subordinate matter.

### Special Nature of Training Required.

It was not long before it was realised that the delicate task of readjusting social dislocations, of promoting co-operation within and without the hospital, required someone of more than ordinary gifts and with a particular training and experience. This realisation eventuated in the creation of the Hospital Almoners' Council, an evolution, it might be said, rather than a creation, as a training committee has been in existence for some time previously. This Council consists of seventy to eighty men and women interested in or working for hospitals and kindred social activities, and carrying on its routine work through an elected Executive Council. Its function is, and always has been, to discover and to train suitable women for this service. It has had to encounter many difficulties and criticisms. With our characteristic British love for muddling through and our ingrained distrust of the expert, it has seemed to many that any ordinary common-sensed person could carry on the job without special training. This is, however, obviously absurd; it is not so that real efficiency is attained, either in the soldier or the social worker. The Hospital Almoners' Council has been driven by experience to extend both the duration and the variety of the training, so that it now occupies a period of two years. Of this period, part is spent in theoretical instruction in economic social theory, along with practical experience in the homes of the poor and in acquiring a knowledge of the various agencies, both public and private, working for the welfare of the citizen. The remainder is spent in gaining experience of the actual duties of an almoner, in one or other of seven hospitals in London, where the student is initiated into the details of dealing with patients and all the many problems and complexities which that involves. The training is supervised individually by an experienced tutor and reports of each student's progress received by the Council from time to time, and on its satisfactory completion a certificate is granted certifying the holder to be fully trained, capable of performing the duties of a hospital almoner.

### Work in the Provinces.

In view of the increasing demand for almoners in the large towns of England and Scotland the Council felt it important that students who wish to take up work in the provinces should be able to obtain experience of conditions in those places. Arrangements have therefore been made with several universities whereby individuals when selected may obtain a considerable part of the qualifying training in such towns.

It is to be seen that the work is many-sided and necessitates considerable organisation. A patient may and often does pass through several departments of the hospital, and information as to social circumstances and home conditions as acquired must be kept available for the various members of the staff concerned. Of late this information is proving valuable in the matter of assessing patients' payments, seeing that most of the hospitals are now considering it necessary to add to their diminishing income by some contribution from those benefited. This additional work naturally falls within the scope of an almoner's duties.

The absorption of so many women in various activities during the war led to a serious shortage of candidates for almoner's training. That temporary difficulty is, however, happily passed; and there are now a number of women in training who in a few months' time will be fully qualified to take up duty—some all the more efficiently, no doubt, because of the special experiences they have passed through.

---

Founders' Day at "The National."—The National Hospital for the Paralysed, Queen Square, kept open house on Tuesday last, when the Founders' Day was celebrated. The order of the day was a service at three o'clock in the chapel, when the Chairman of the hospital, Sir Frederick Macmillan, read the lesson—St. Paul on the gift of Charity—and the Hon. and Rev. Canon J. G. Adderley, M.A., Rector of St. Paul's, Covent Garden, gave an address. Later tea was served in the Board-room, where the big open fire and large tables laid for tea gave a warm welcome to the visitors, amongst whom were H.R.H. the Duchess of Albany, attended by Miss Heron Maxwell and many supporters of the hospital. The wards were thrown open for inspection, and visitors were conducted by members of the staff over the pensioners' wards and exercise-room, the annexe for discharged soldiers and sailors, and the out-patient and electrical department.

